# Novel Mixed Matrix Membranes Based on Polyphenylene Oxide Modified with Graphene Oxide for Enhanced Pervaporation Dehydration of Ethylene Glycol

**DOI:** 10.3390/polym14040691

**Published:** 2022-02-11

**Authors:** Mariia Dmitrenko, Anastasia Chepeleva, Vladislav Liamin, Anton Mazur, Konstantin Semenov, Nikolay Solovyev, Anastasia Penkova

**Affiliations:** 1 St. Petersburg State University, 7/9 Universitetskaya nab., 199034 Saint Petersburg, Russia; chepeleva1999@yandex.ru (A.C.); lyamin.vlad.322@gmail.com (V.L.); a.mazur@spbu.ru (A.M.); a.penkova@spbu.ru (A.P.); 2 Pavlov First Saint Petersburg State Medical University, L’va Tolstogo Ulitsa 6-8, 197022 Saint Petersburg, Russia; semenov1986@yandex.ru; 3 Institute of Technology Sligo, Ash Lane, F91 YW50 Sligo, Ireland; solovyev.nikolay@itsligo.ie

**Keywords:** polyphenylene oxide, mixed matrix membrane, graphene oxide, pervaporation, ethylene glycol dehydration

## Abstract

Ethylene glycol (EG) is widely used in various economic and industrial fields. The demand for its efficient separation and recovery from water is constantly growing. To improve the pervaporation characteristics of a poly(2,6-dimethyl-1,4-phenylene oxide) (PPO) membrane in dehydration of ethylene glycol, the modification with graphene oxide (GO) nanoparticles was used. The effects of the introduction of various GO quantities into the PPO matrix on the structure and physicochemical properties were studied by Fourier-transform infrared (FTIR) and nuclear magnetic resonance (NMR) spectroscopies, scanning electron (SEM) and atomic force (AFM) microscopies, thermogravimetric analysis (TGA), swelling experiments, and contact angle measurements. Two types of membranes based on PPO and PPO/GO composite were developed: dense membranes and supported membranes on a fluoroplast substrate (MFFC). Transport properties of the developed membranes were evaluated in the pervaporation dehydration of EG in a wide concentration range (10–90 wt.% and 10–30 wt.% water for the dense and supported membranes, respectively). The supported PPO/GO(0.7%)/MFFC membrane demonstrated the best transport properties in pervaporation dehydration of EG (10–30 wt.% water) at 22 °C: permeation flux ca. 15 times higher compared to dense PPO membrane—180–230 g/(m^2^·h)), 99.8–99.6 wt.% water in the permeate. The membrane is suitable for the promising industrial application.

## 1. Introduction

Ethylene glycol (EG) is widely used in the chemical, textile, automotive, and electrical industries [1]. In particular, the applications include the synthesis of polyester fibers, polyethylene glycol, polyethylene terephthalate, polyester, cellophane, polyurethane, and other products, and use in antifreeze, brake fluids, anti-icing fluids, etc. In most involved processes, wastewater with EG is formed. High-purity EG represents a valuable organic matter, so it is justified to extract and regenerate it from the wastewater. It is also worth noting that EG production is usually carried out by hydrolysis of ethylene oxide in the presence of excess water [2]. The enriched result by distillation is a 70–80 wt.% aqueous solution of ethylene glycol, which requires an additional dehydration step. The further concentration of EG by distillation becomes economically non-feasible, arduous, and energy-intensive, due to the lower water content in the vapor phase and a very high boiling point (197.3 °C) of ethylene glycol [2]. However, the use of pervaporation with the correct selection of a membrane with the tailored characteristics makes it possible to easily and energy-efficiently solve the problem of EG recovery from water [3].

Pervaporation, as a membrane process, is related to sustainable possesses; it is an energy-efficient, cost-effective, environmentally friendly, and waste-free method for separating liquid mixtures with low molecular weight substances. A distinctive characteristic of this separation method is the phase transition from liquid to vapor under a driving force, providing high efficiency of separation of azeotropic and isomeric mixtures, close-boiling, and thermally unstable substances [4]. The mechanism of pervaporation consists of three main stages according to the “solubility–diffusion” mechanism and does not depend on the vapor–liquid equilibrium [5]. This method as a diffusive membrane process also allows the sensitive evaluation of the contribution of membrane modification [6]. Nowadays, the majority of research on EG dehydration by pervaporation has focused on the use of polymeric membranes based on a polymer of intrinsic microporosity (PIM-1) [7], polyvinyl alcohol (PVA) [8], polyvinylamine (PVAm)–PVA [9], chitosan [10], polyelectrolyte complexes [11,12], polybenzimidazole/polyetherimide [13], etc. In [7], the PIM-1 membrane was prepared and applied for EG purification from the water/methanol mixtures by pervaporation. It demonstrated a high permeation flux but a moderate separation factor: 50 g/(m^2^·h) permeation flux and 39.11 separation factor in pervaporation dehydration of EG (10.33 wt.% water) at 30 °C, and 211 g/(m^2^·h) permeation flux and 12.67 separation factor in pervaporation separation of EG–methanol mixture (11.45 wt.% methanol) at 30 °C. The transport properties of the PVA membrane for pervaporation dehydration of EG were improved by the modification with zeolite 4A (5 wt.%) and the development of a supported membrane on a polypropylene microfiltration membrane (substrate) [8]. It was shown that the zeolite 4A addition increased the separation factor and improved permeation flux, with 2.65 kg/(m^2^·h) and a separation factor of 1972 in pervaporation dehydration of EG (20 wt.% water) at 70 °C. The supported membranes with a thin selective layer based on a PVAm–PVA blend on a microporous polysulfone (PS) substrate were improved by the modification with carbon nanotubes (CNTs) [9]. The addition of CNTs into the matrix significantly improved the membrane performance only at low feed water concentrations. The PVAm–PVA/CNTs (2 wt.%) membrane had the optimal transport properties: a permeation flux of 146 g/(m^2^·h) and a separation factor of 1156 in EG dehydration (1 wt.% water in the feed) at 70 °C. In [10], the developed supported chitosan/PS membrane had the following parameters: a permeation flux of 0.3 kg/(m^2^·h) and a separation factor of 109 (92.4 wt.% water in the permeate) in pervaporation dehydration of EG (10 wt.% water) at 35 °C. A supported membrane based on polyelectrolyte complex nanoparticles (PEC NPM)-graphene oxide (GO) composites on a PS substrate was investigated for pervaporation dehydration of EG [11]. The best membrane PEC NPM/GO (3 wt.%)/PS had ca. 1.5 times lower permeation flux (961 g/(m^2^·h)) and ca. 2.5 times higher separation factor (1191) compared to the pristine membrane in dehydrating 10 wt.% water/EG mixtures at 60 °C. However, these membranes were studied with dehydration only up to 20 wt.% water in the feed. In [12], polyelectrolyte polyethylenimine/poly(acrylic acid) (PEI/PAA) membranes prepared by layer-by-layer self-assembly (seven double layers) on top of an interfacially polymerized polyamide (PA) substrate were studied for pervaporation dehydration of EG, ethanol, and isopropanol. The pervaporation experiments were carried out only above 20 wt.% water in the feed. The PEI/PAA/PA membrane demonstrated a permeation flux of ca. 5–15 g/(m^2^·h) and a separation factor of ca. 570–410 in pervaporation dehydration of EG (2–20 wt.% water) at 22 °C. Polybenzimidazole/polyetherimide (PBI/PEI) dual-layer hollow fiber membranes were studied by the pervaporation dehydration of EG under various conditions [13]. They demonstrated a separation factor of 1763 and a permeation flux of 115 g/(m^2^·h) in pervaporation dehydration of EG (20 wt.% water) at 60 °C. Although many polymeric membranes were proposed and studied for the pervaporation dehydration of EG, further development of more efficient membranes is still required, maintaining the trade-off of high permeation flux and high selectivity, as well as high stability for purifying EG with high water content in the feed (above 20 wt.%).

Among these membrane polymeric materials, poly(2,6-dimethyl-1,4-phenylene oxide) (PPO) deserves special attention. PPO is an aromatic glassy polymer with high mechanical and thermal stability, resistance to chemical agents, and high permeability to gases [14]. Due to the ease of movement of phenyl rings and large free volume between the polymer chains, this polymer is actively used as a membrane material for diffusion membrane processes—gas separation [15,16,17] and pervaporation [18,19,20,21,22]. However, in spite of its potential, PPO was investigated only in a few studies which addressed the pervaporation regeneration of EG from water [23] and methanol [24,25]. For the separation of EG/water mixtures, the improvement of PPO membranes was achieved by the modification with heteroarm stars (HAS), consisting of a center fullerene C_60_, six arms of nonpolar polystyrene, and six arms of polar poly-tert-butyl methacrylate [23]. A dense PPO/HAS (5 wt.%) membrane with the thickness of ca. 40 μm was tested in the pervaporation dehydration of EG (5–12 wt.% water) at 50 °C and demonstrated the optimal transport properties: ca. 0.003–0.013 kg/(m^2^·h) permeation flux and 32,500–10,000 separation factor (ca. 99.9 wt.% water in the permeate). However, for the promising application of PPO membranes in industrial processes of EG dehydration, highly efficient supported membranes with a thin selective layer on porous substrates are required. Thus, the selection of another modifier and the creation of supported PPO membranes are a promising direction to improve the transport properties enhancing EG dehydration.

In the present work, the improvement of PPO membrane performance for dehydration of EG was achieved by the modification with one of the most promising carbon nanoparticles—graphene oxide (GO). GO is obtained by oxidation of graphite, and it is possible to obtain various GO structures depending on the applied method [26]. Graphene oxide has unique structural properties, good dispersion in polymer matrices, and functional oxygen-containing groups. However, at present, GO still has limited use as a nanotailored material, due to a low adsorption capacity and weak interfacial interaction [27]. One of the most promising ways is to use GO as a modifier of polymeric membranes to enhance their properties and sustainability in gas separation and water purification [28,29,30,31]. However, to the best of our knowledge, there are no works on the use of mixed matrix PPO membranes with GO modification in pervaporation [32]. The introduction of a carbon nanomodifier such as GO into a PPO membrane would significantly affect the hydrophilic–hydrophobic balance of the surface, change the crystallinity and morphology of the polymer matrix, and change the free volume between polymer chains, causing significantly enhanced transport properties in pervaporation [6].

Thus, the aim of the study was to improve the pervaporation characteristics of a membrane based on poly(2,6-dimethyl-1,4-phenylene oxide) in the dehydration of ethylene glycol by the modification with graphene oxide nanoparticles and the developing of high-performance supported membranes for promising industrial applications. The effect of the introduction of the various GO quantities into the polymer matrix on the structural, physicochemical, and transport properties of the PPO-based membranes was investigated. Two types of membranes were developed: dense and supported on a fluoroplast substrate (MFFC). The supported membranes with a thin selective layer deposited onto a porous MFFC substrate were created to significantly increase the membrane permeability and for the potential implementation in industry. The structure and physicochemical properties of the developed membranes were studied by spectroscopic (FTIR and NMR) and microscopic (SEM and AFM) techniques, thermogravimetric analysis (TGA), swelling experiments, and contact angle measurements. Transport properties of the PPO-based membranes were tested in the pervaporation dehydration of EG in a wide concentration range (10–90 wt.% and 10–30 wt.% water in the feed for the dense and supported membranes, respectively).

## 2. Materials and Methods

### 2.1. Materials

Poly(2,6-dimethyl-1,4-phenylene oxide) (PPO, 1.06 g/mL at 25 °C, Sigma-Aldrich, St. Petersburg, Russia) was used as a membrane matrix. Graphene oxide (GO, Fullerene Technologies, St. Petersburg, Russia) synthesized from graphite by an oxidation reaction using a modified Hummers and Offeman method [33] was used as a modifier of the PPO membranes. Commercial porous hydrophobic membrane MFFC (Vladipor, Vladimir, Russia) based on fluoroplast F42L was used as a support for the preparation of the supported PPO-based membranes. Chloroform (CHCl_3_, purity ≥ 99.1 wt.%), ethylene glycol (EG, purity ≥ 99.9 wt.%), and methanol (MeOH, purity ≥ 99.5 wt.%) were purchased from Vekton (St. Petersburg, Russia) and used without additional treatment.

### 2.2. Membrane Preparation

The preparation of PPO/GO composites was carried out by solid-phase synthesis [34]. The pre-determined amount of polymer powder was ground with the calculated quantity of GO (0.1, 0.3, 0.5, 0.7, 0.9 wt.% with respect to the polymer weight). The obtained composite was dissolved in chloroform under constant stirring for 3 h at ambient temperature to obtain 8 wt.% PPO solution with the consequent ultrasonic treatment for 30 min at ambient temperature for degassing the polymer solution [35]. Dense PPO and PPO/GO membranes were formed by solvent evaporation technique: pouring pre-determined PPO solution or composite onto the cellophane surface fixed on a hollow steel ring, drying in an oven at 40 °C for 12 h to remove the solvent, and then separating the membrane from the cellophane following [36]. The thickness of the dense PPO-based membranes measured by a micrometer was equal to 35 ± 5 μm.

**Figure 1:**
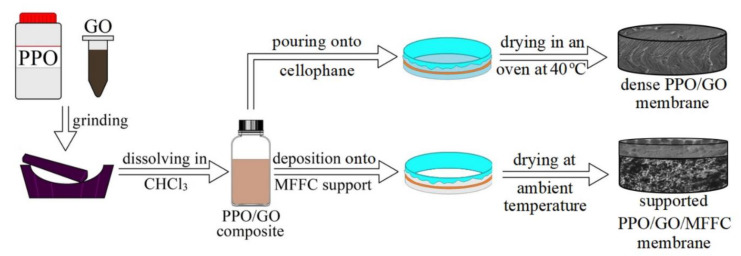
The preparation scheme of the PPO-based membranes.

Supported membranes were prepared by the deposition of the prepared polymer solution and composite onto commercial porous MFFC membrane to form a thin selective layer based on the PPO and PPO/GO (0.7%) composite in the following manner. The MFFC substrate was fixed on a hollow steel ring, the PPO solution or composite were cast onto the substrate from the side of the ring, and the excess casting solution was removed. Then, the membranes were left to dry at ambient temperature for 24 h to evaporate the solvent. The preparation scheme of the dense and supported membranes is summarized in Figure 1.

### 2.3. Pervaporation

**Figure 2:**
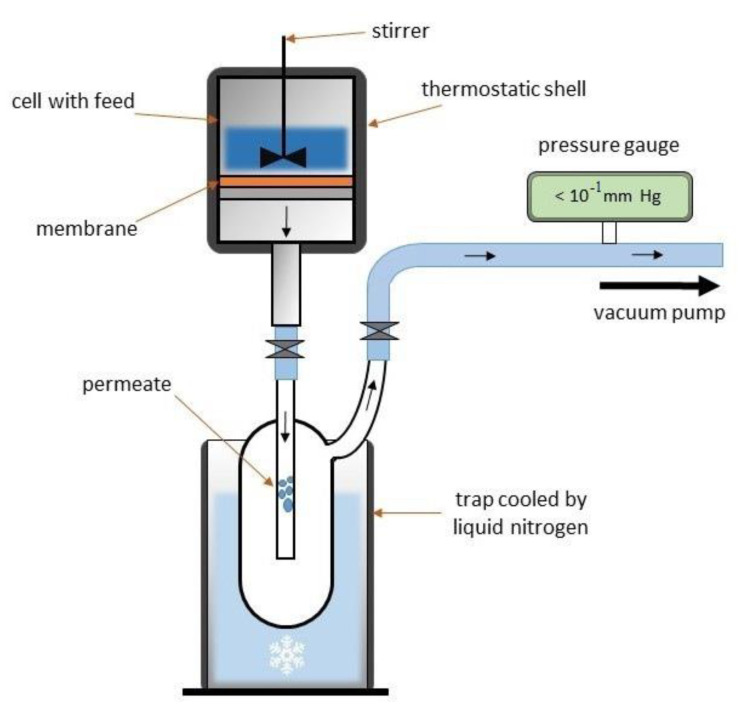
The scheme of the pervaporation setup.

The PPO-based membranes were evaluated in pervaporation separation of ethylene glycol/water mixture (10–90 wt.% water) in a steady-state cell at 22 °C with a membrane area of 9.61 cm^2^ and <10^−1^ mm Hg downstream pressure [37,38]. The scheme of pervaporation setup is demonstrated in Figure 2.

The transport properties of the PPO-based membranes are presented in terms of permeation flux, component permeances, water content in the permeate, and separation factor.

Permeation flux (*J*) of the membranes was calculated according to the following equation [39]:

(1) $$J=\frac{W}{A\cdot t},$$

where *W* is the weight of permeate collected in a trap cooled by liquid nitrogen (kg), *A* is the membrane area (m^2^), and *t* is the time of permeate collection (h).

The separation factor (*β*) was calculated according to the following equation [40]:

(2) β=yiyjxixj,

where *y_i_* and *y_j_* are the *i* and *j* component contents in the permeate, and *x_i_* and *x_j_* are the *i* and *j* component contents in the feed.

The component permeances (*P/l*) (a component flux normalized for driving force) were calculated according to the following equation [40]:

(3) $$\frac{P}{l}=\frac{j_{i}}{p_{i_{f}}-p_{i_{p}}},$$

where *j_i_* is the partial component flux, *l* is the membrane thickness, and $p_{i_{f}}$ and $p_{i_{p}}$ are vapor pressures of components in the feed and the permeate, respectively. The permeance was calculated in common gas permeation units (GPU) according to Baker et al. [40].

The permeate was analyzed by gas chromatography with a Chromatek Crystal 5000.2 chromatograph (Chromatec, Nizhny Novgorod, Russia) equipped with a Hayesep R column (Chromatec, Nizhny Novgorod, Russia). Pervaporation experiments were carried out at least three times for each membrane type; then, average values of the parameters were calculated and taken for analysis. The transport parameters’ mean accuracy of the PPO-based membranes was ±0.5% for water content in the permeate, and ±10% and ±15% for the permeation flux of the dense and supported membranes, respectively.

### 2.4. Fourier-Transform Infrared Spectroscopy (FTIR)

The structural changes during the modification of PPO with GO were studied by FTIR spectroscopy using an IRAffinity-1S spectrometer (Shimadzu, St. Petersburg, Russia) and an attenuated total reflectance (ATR) accessory (PIKE Technologies, St. Petersburg, Russia) in the range of 500–4000 cm^−1^ at 25 °C.

### 2.5. Nuclear Magnetic Resonance (NMR)

NMR study of PPO-based membranes was carried out using a Bruker Avance III 400 WB NMR spectrometer (Bruker, Bremen, Germany) with a CP/MAS probe of 4 mm and a magnetic field of 9.4 T. Larmor frequency for nuclei ^13^C was 100.64 MHz. Liquid tetramethylsilane (TMS) was applied as an external reference for ^13^C nuclei.

### 2.6. Scanning Electron Microscopy (SEM)

The morphology of the inner and surface structure of PPO-based membranes was investigated by SEM using a Zeiss Merlin SEM (Carl Zeiss SMT, Oberhochen, Germany) at a low accelerating voltage (1 kV) and the low electron beam current (100 pA) preventing surface modification and charging.

### 2.7. Atomic Force Microscopy (AFM)

The surface of the developed PPO-based membranes was studied by AFM with an NT-MDT NTegra Maximus atomic force microscope (NT-MDT Spectrum Instruments, Moscow, Russia) with standard silicon cantilevers (15 N·m^−1^ rigidity) in the tapping mode.

### 2.8. Thermogravimetric Analysis (TGA)

The thermochemical stability of the PPO-based membranes was investigated by TGA using a Thermobalance TG 209 F1 Libra (Netzsch, Leuna, Germany) under argon atmosphere and in a temperature range of 30–950 °C with a 10 K/min heating speed.

### 2.9. Contact Angle Measurements

Contact angles of water were measured by the sessile drop method with a Goniometer LK-1 instrument (Ltd. NPK Open Science, Krasnogorsk, Russia) to assess the hydrophilic–hydrophobic properties of the surface of the PPO-based membranes. To analyze contact angle results, the DropShape software (Ltd. NPK Open Science, Krasnogorsk, Russia) was used.

### 2.10. Swelling Experiments

The swelling degree of the dense PPO-based membranes in water and EG was measured by a gravimetric method at 22 °C. The weighed membranes were immersed in the corresponding liquids and weighed daily until the constant swelling weight. The swelling degree (*S*) of the PPO-based membranes was calculated as follows:

(4) $$S=\frac{m_{s}-m_{0}}{m_{0}},$$

where *m*_0_ is the initial membrane weight (g), and *m_s_* is the swollen membrane weight (g).

## 3. Results

This section is divided into four main parts. Section 3.1 is devoted to the investigation of transport properties of the dense and supported PPO and PPO/GO membranes in the pervaporation dehydration of EG. Section 3.2 is dedicated to the study of structural and physicochemical properties of the developed PPO-based membranes to explain and describe the obtained transport characteristics and mass transfer of the components through the membranes. In Section 3.3, the comparison of pervaporation performance of membranes based on the PPO/GO (0.7%) composite with literature-described polymeric membranes is presented.

### 3.1. Transport Properties of the PPO-Based Membranes

**Figure 3:**
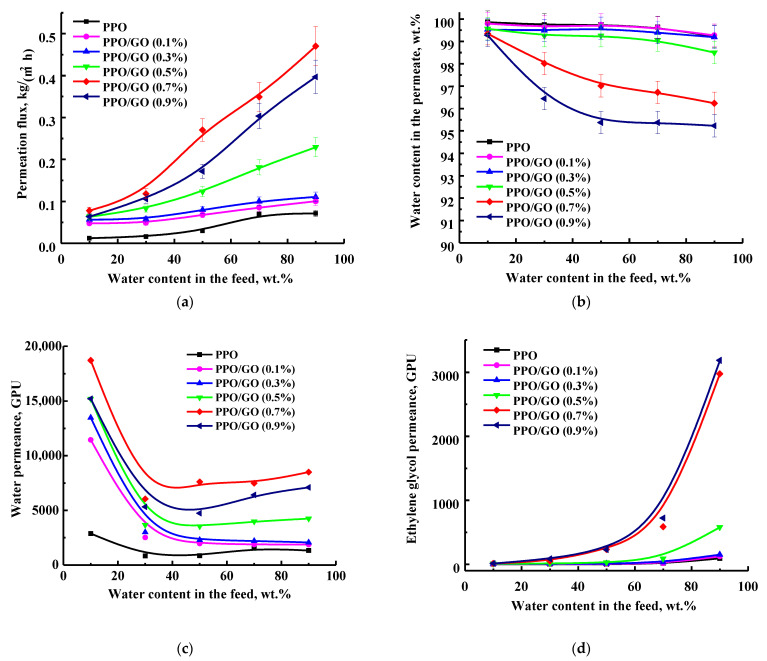
The dependence of (**a**) permeation flux, (**b**) water content in the permeate, and (**c**,**d**) component permeances on the water content in the feed in pervaporation separation of ethylene glycol/water mixture (10–90 wt.% water) for the dense PPO and PPO/GO membranes.

To study the effect of modification with graphene oxide, PPO membranes with different modifier quantities (0.1, 0.3, 0.5, 0.7, and 0.9 wt.%) were tested in the pervaporation dehydration of EG in a wide concentration range (10–90 wt.% water), also assessing the effects of feed composition. Figure 3 displays transport characteristics of the developed membranes in terms of permeation flux, water content in the permeate, and component permeances.

Although PPO polymer has phenyl groups and is hydrophobic, PPO-based membranes are known to transmit small molecules (for example, water) in vacuum pervaporation [41]. As pervaporation is described by a solubility–diffusion mechanism [42], one of the most robust approaches to evaluate the interaction between PPO and components of the feed (water and EG) is the use of Hansen’s solubility parameters. According to the theory of solubility, a smaller difference in the solubility parameters of the polymer and the liquid contributes to stronger interaction of this liquid with the polymer, causing a higher sorption degree of the component in the polymer [37]. The data on solubility parameters of water (49.6 (J/cm^3^)^1/2^), EG (32.9 (J/cm^3^)^1/2^), and PPO (18.2 (J/cm^3^)^1/2^) are presented in [23]. A smaller difference between solubility parameters of the EG and PPO (∆ = 14.7 (J/cm^3^)^1/2^) is shown, which suggests a higher EG solubility and sorption in PPO membranes than in water. The increased membrane swelling (sorption) in EG was also confirmed by swelling data in Section 3.2.1. The mechanism of mass transfer of the EG/water mixture through the PPO-based membranes may be described as follows: EG initially interacts with PPO through hydrogen bonds due to its higher solubility in the membrane and creates transport channels; then, water penetrates into the channels, the size of which is commensurate with the kinetic diameter of a water molecule [41]. All developed PPO-based membranes were confirmed to be highly selective to water (Figure 3b). The obtained data demonstrate that the permeation flux and water content in the permeate of both pristine PPO and modified PPO/GO membranes are a function of the water content in the feed: with the rise in water in the feed, the water content in the permeate slightly decreases, and the permeation flux of all membranes increases [43].

The introduction of GO into the PPO matrix improved the permeation flux with a slight decrease in selectivity with respect to water, compared to the unmodified PPO membrane (Figure 3a,b). It is also worth noting that the observed changes propagated with an increase in the GO concentration up to 0.7 wt.% in the membrane, except for the membrane with 0.9 wt.% GO. The PPO/GO (0.9%) membrane had lower permeation flux and water content in the permeate compared to the PPO/GO (0.7%) membrane. It may be related to the agglomeration of GO particles in the PPO matrix, causing the hindered mass transfer of the components through the membrane, which decreased the membrane’s permeability. Moreover, the agglomeration of GO particles led to the formation of defects in the membrane due to selective exfoliation on the interface between the polymer matrix and agglomerated nanoparticles phase [24], which initiated additional transport channels for both water and EG, reducing the selectivity of the membrane [44]. The improvement in permeability of the GO-modified membranes is associated with a change in the inner and surface structure: an increase in the roughness and amorphous phase of the matrix morphology and in the surface roughness and its hydrophilization during the modification process (confirmed by NMR, SEM, AFM, and contact angle data—see below). However, with an increase in water content in the feed, the membranes swelled more, leading to plasticization of the membrane and increasing the free volume between polymer chains, resulting in simultaneous penetration of EG and water and a decrease in the selective properties (Figure 3b). The optimal transport characteristics were exhibited by the PPO/GO (0.7%) membrane: the highest permeation flux (≥5 times higher compared to the pristine PPO membrane) with a slight decrease in selective properties (99.3–96.2 wt.% water in the permeate), while water content in the permeate for PPO membrane reached 99.9–99.2 wt.%.

The transport properties of the PPO-based membranes were considered in terms of component permeances (a component flux normalized for the driving force), related to the intrinsic properties of the membranes (Figure 3c,d). After normalizing for the driving force, it was observed that the dependence of permeation flux from water concentration in the feed (Figure 3a) was mostly related to the changes in water and EG vapor pressure (driving force) with the concentration [40]. An increase in the feed water content means a decrease in the EG concentration, resulting in the enhanced driving force for the water penetration through the membrane and depressed driving force for the EG penetration. Water permeance for all membranes was significantly higher than the EG permeance, due to the special mass transfer mechanism manifesting for the PPO-based membranes [3]. Thus, the permeation flux was dominated by the flux of water. However, in contrast to permeation flux (Figure 3a), water permeance for the modified membranes was found to decrease. The water permeance was high at a low concentration (10 wt.% water in the feed) but decreased to a plateau value at the concentration above 30 wt.% water in the feed (Figure 3c). It may be related to a significant increase in fugacity of water with feed concentration compared to EG [45]. A similar trend for the permeation flux and component permeabilities (alternative to permeances) was considered for the separation of acetic acid–water mixtures using mixed matrix copolymer membranes in [45]. The PPO/GO (0.7%) membrane had the highest values of water permeance, which is in agreement with the highest permeation flux (Figure 3a). However, this membrane was also characterized by high values of EG permeance, which may be associated with the swelling of the membrane due to the modification (confirmed below by swelling data) and its plasticization in the feed.

**Figure 4:**
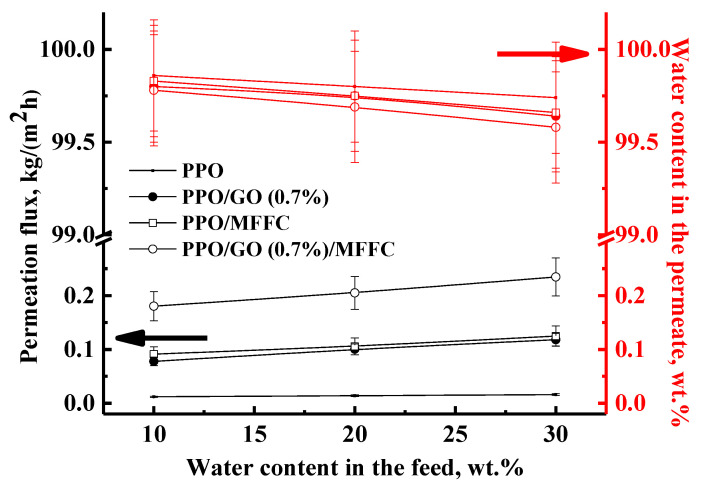
The dependence of permeation flux and water content in the permeate on the water content in the feed in pervaporation separation of ethylene glycol/water mixture (10–30 wt.% water) for the dense and supported membranes based on the PPO and PPO/GO (0.7%) composite.

For the promising industrial application in EG regeneration of the PPO/GO (0.7%) membrane with optimal transport properties, the development of a supported membrane was carried out by the deposition of a thin selective layer based on PPO and PPO/GO (0.7%) composite onto a porous MFFC substrate. All commercial industrial membranes are supported due to better mechanical strength and enhanced permeability. It was found that energy consumption for EG dehydration using membrane technology—pervaporation—can be significantly reduced if the concentration of EG in the mixture exceeds 70 wt.% [1,2,46]. Thus, transport properties of the obtained supported PPO/MFFC and PPO/GO (0.7%)/MFFC membranes were tested in pervaporation dehydration of EG (10–30 wt.% water) and compared with the dense membranes (Figure 4).

The development of the supported membranes led to the increased permeation flux by ca. 8 and 2 times for the membranes based on the PPO and PPO/GO (0.7%) composite, respectively. Moreover, high selectivity with respect to water (99.8–99.7 wt.% and 99.8–99.6 wt.% water in the permeate, respectively) was maintained. The reason for the permeation flux enhancement was related to a significant decrease in the thickness of the dense selective layer of the membrane (confirmed by SEM below). A smaller degree of increase in permeation flux for the modified membrane may be associated with the presence of GO nanoparticles in the thin selective layer, which can hinder the mass transfer of the components through the membrane [34]. The developed supported PPO/GO (0.7%)/MFFC membrane (modified with 0.7 wt.% GO) had the best transport properties (the highest permeation flux, which is ca. 15 times higher compared to the dense pristine PPO membrane, maintaining high selectivity) for the pervaporation dehydration of EG (10–30 wt.% water), and is promising for the industrial application for EG regeneration.

### 3.2. The Characterization of PPO-Based Membranes

#### 3.2.1. The Study of the Dense Membranes

**Figure 5:**
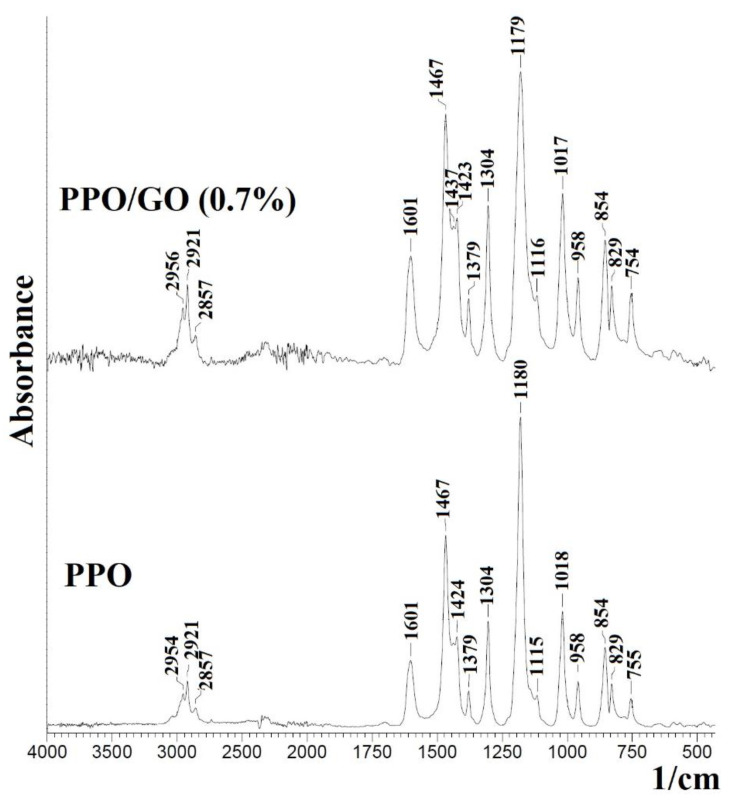
FTIR spectra of the PPO and PPO/GO (0.7%) membranes.

To study the effect of modification with GO, the structural characteristics of the developed PPO-based membranes were investigated by FTIR and NMR spectroscopies, SEM, and AFM methods. FTIR spectra of the PPO and PPO/GO (0.7%) membranes, with optimal transport properties, are presented in Figure 5.

The FTIR spectrum of pristine PPO membrane demonstrates the characteristic absorption bands. The peak at 2954 cm^−1^ corresponds to vibrations of aromatic C–H bonds. The peaks at 2921 and 2857 cm^−1^ belong to asymmetric and symmetric vibrations of –CH_2_ groups. The peaks at 1601 and 1467 cm^−1^ correspond to the vibrations of C=C and C–H of the benzene ring, respectively, and the peaks at 1180 and 1304 cm^−1^ refer to symmetric and asymmetric vibrations of the C–O bond [47]. It should be noted that the introduction of 0.7 wt.% GO into the PPO matrix did not significantly change the band position and intensity. In the spectrum of the modified PPO/GO (0.7%) membrane, characteristic peaks of GO are not observed: the wide peak around 3440 cm^−1^ related to the –OH stretching of the carboxylic group, the peak at 1718 cm^−1^ attributed to the C=O stretching of the carboxylic groups, the peak at 1630 cm^−1^ corresponded to the C=C stretch of the aromatic domain of graphene, etc. [48,49]. These bands may be invisible due to the low GO content in the membrane matrix and indicate weak interactions between the polymer and the particles of the modifier.

**Figure 6:**
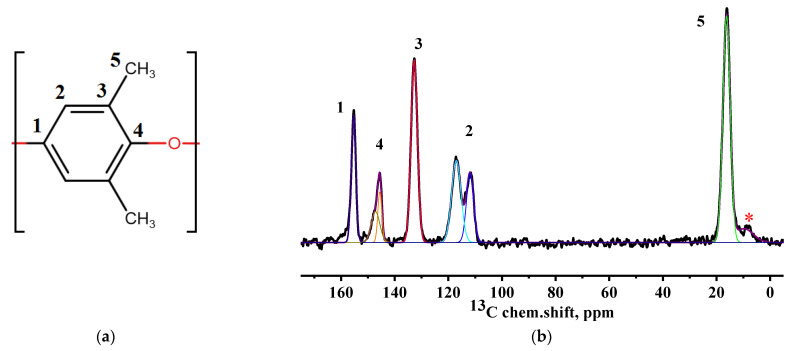
(**a**) Schematic representation of a PPO monomer with numbered nonequivalent carbon atoms and (**b**) ^13^C NMR spectrum of the pristine PPO membrane.

The structural changes in the GO-modified PPO membranes were confirmed by NMR spectroscopy. Figure 6 demonstrates a schematic representation of a PPO monomer with numbered nonequivalent carbon atoms and the ^13^C NMR spectrum of the pristine PPO membrane.

**Figure 7:**
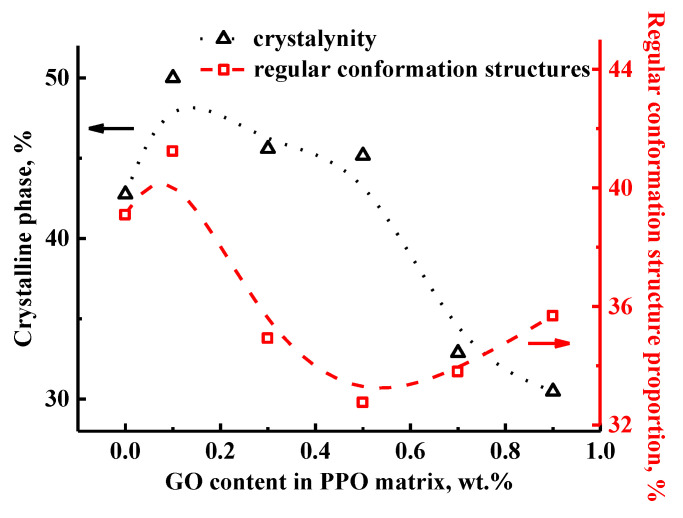
The dependences of crystalline phase and regular conformation structure proportion on the GO content in the PPO matrix.

The spectrum shows the presence of five allowed spectral components, each of which corresponds to carbon atoms in different nonequivalent positions (Figure 6a). There is a weak peak (asterisk in Figure 6b) near the line, corresponding to the methyl groups of PPO. This peak is a rotation satellite (SSB) from the line at position 3 of the polymer unit. In addition, it can be seen that the spectral line, related to the carbon atoms in position 4, is divided into two weakly resolved components. This may be related to the presence of regions with regular conformational packing of polymer units in the polymer structure [50]. Since the component (at 112 ppm) has a smaller width, it can be assumed to correspond to the conformationally ordered phase of the polymer. The change in the content of the conformationally ordered phase in the samples during the modification with GO is possible to estimate by evaluating the ratio of the integral areas of the two components in the spectrum (Figure 7). In turn, the spectral line, corresponding to the atoms in the position 4, has an inhomogeneously broadened shape. This may indicate the presence of two unresolved components. The presence of two components at carbon atoms, which are connected with the interlink oxygen atom, can be associated with the presence of an NMR crystalline phase in the volume of the studied membranes. As in the previous case, the line at about 145.5 ppm is characterized by a smaller width, which indicates that it belongs to the atoms of the ordered phase. The content of the NMR crystalline phase in the developed membranes was also evaluated by the estimation of the ratio of the integral areas of these two lines (Figure 7).

The obtained data demonstrate that the PPO/GO (0.1%) membrane with a minimum content of the modifier has a slight increase in the proportion of regular conformation structure compared to the pristine PPO membrane. With a further increase in the GO concentration in the membranes, the amount of the conformationally ordered phase, at first, decreases monotonically and reaches a minimum at a GO quantity of 0.5 wt.%, and then a slight increase is observed. This effect indicates that the modification changes the conformation of the polymer chains with a more uneven distribution in the volume. This forms a larger free volume between the PPO chains, reflecting the improved permeability of the modified membranes. For the NMR crystalline phase, another trend of changes is observed: for the PPO membranes with 0.1–0.5 wt.% GO, an insignificant decrease in the amount of the crystalline phase is detected. At the same time, the rise in GO content from 0.7 to 0.9 wt.% in the PPO matrix led to a sharp drop in the content of the NMR crystalline phase. This indicates that the introduction of GO into the PPO matrix forms a membrane with a more amorphous structure, resulting in an increased permeation flux for the modified membranes during EG dehydration (Figure 3a). The NMR data are in agreement with transport membrane characteristics (see above) and the SEM data, described below.

**Figure 8:**
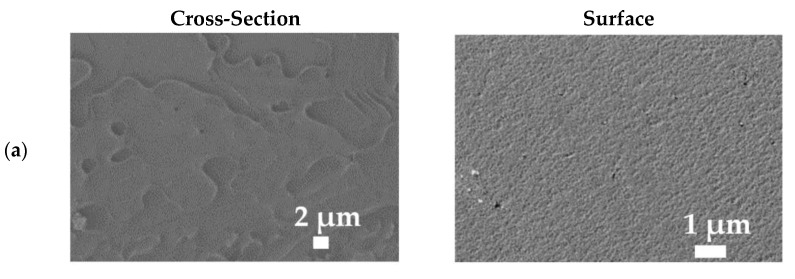

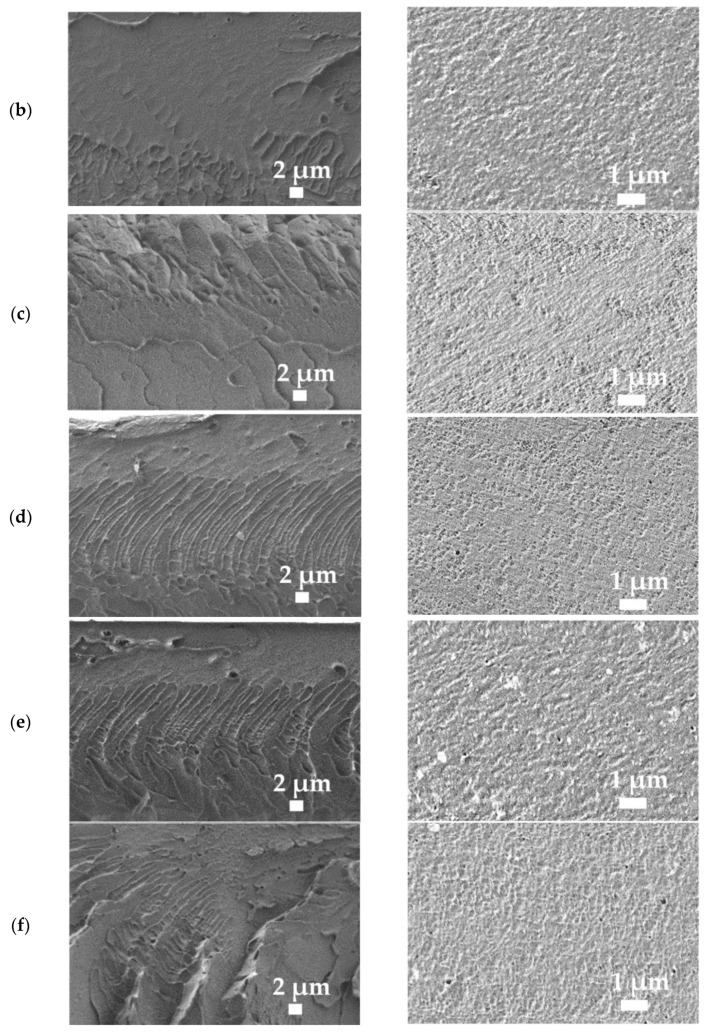
SEM cross-sectional and surface micrographs of the dense (**a**) PPO, (**b**) PPO/GO (0.1%), (**c**) PPO/GO (0.3%), (**d**) PPO/GO (0.5%), (**e**) PPO/GO (0.7%), and (**f**) PPO/GO (0.9%) membranes.

The morphology of the dense PPO-based membranes was studied by SEM; significant changes were observed even at low GO concentrations in the membrane matrix (Figure 8).

The presented micrographs demonstrate a uniform surface structure with rounded cavities and a cross-sectional structure with uniform plastic deformations of the unfilled PPO membrane (Figure 8a). The introduction of GO into the polymer matrix changed these deformations, which became significant and stronger with the increase in the modifier content. The modification with the lowest GO content (0.1 wt.%) slightly altered the cross-section, but formed a surface with a rougher structure (a ridge-and-valley structure) [51] and increased the number of rounded cavities. The formation of more cavities with a larger size may be related to the aggregation of the modifier particles and the phase separation of the PPO and the GO [44]. With the increase in GO concentration (from 0.3 to 0.9 wt.%), the plastic deformations of the cross-section and the surface roughness with rounded cavities increased significantly. The maximum roughest cross-sectional structure and number of cavities and the GO particles on the surface were observed for the PPO/GO (0.9%) membrane, which was consistent with the maximum surface roughness values (confirmed below by AFM data). It is worth clarifying that the GO particles were not visible in the cross-sectional structure of all modified membranes, which could indicate their uniform dispersion in the polymer matrix [51,52].

**Figure 9:**
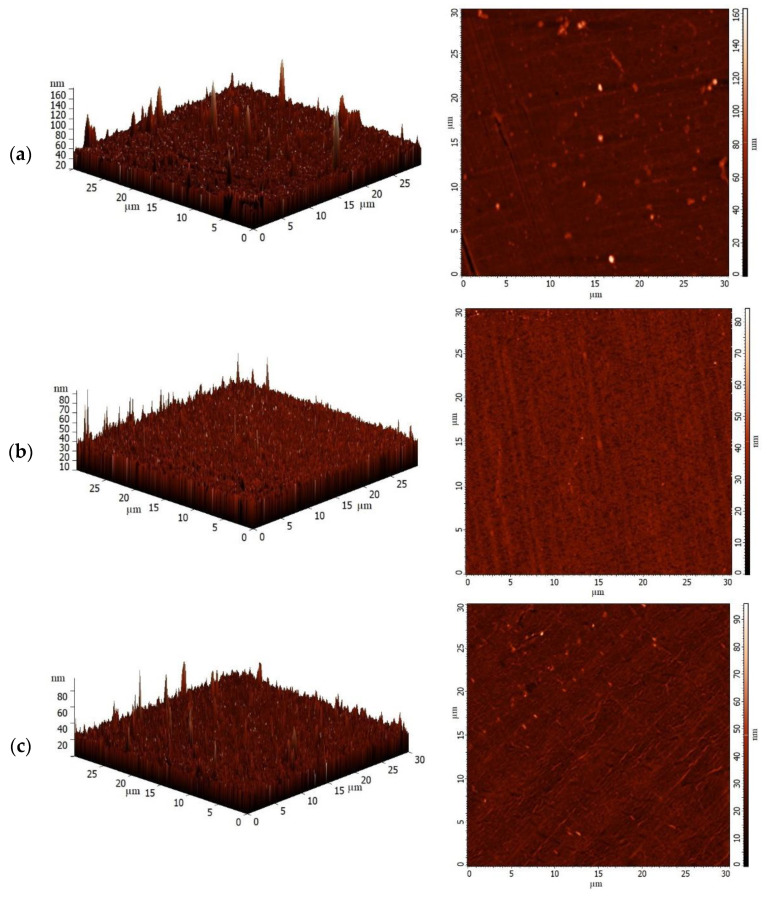

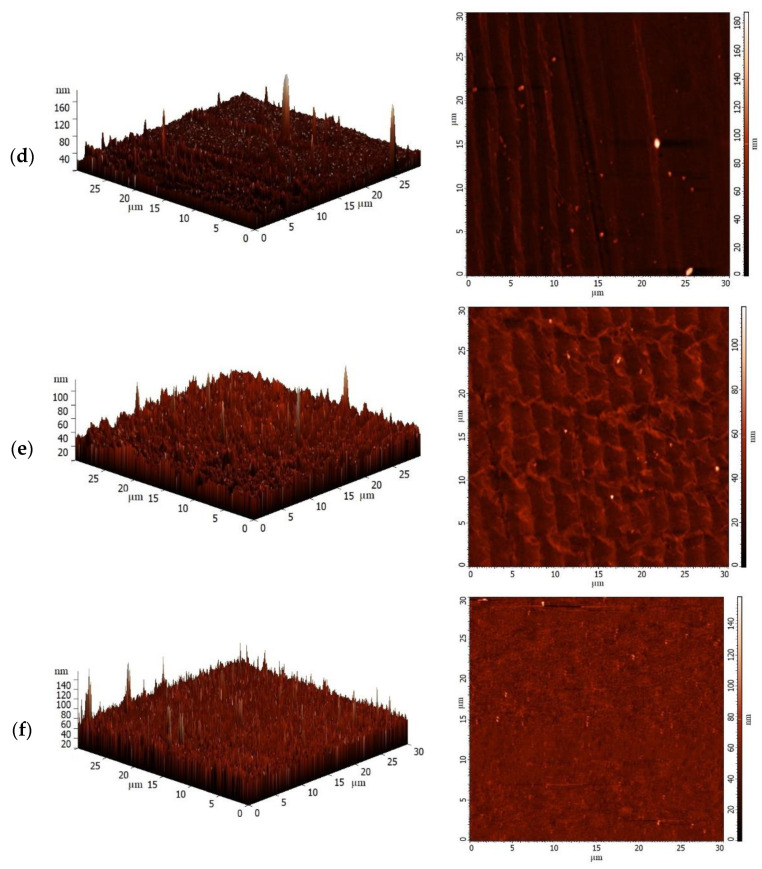
AFM images of the dense (**a**) PPO, (**b**) PPO/GO (0.1%), (**c**) PPO/GO (0.3%), (**d**) PPO/GO (0.5%), (**e**) PPO/GO (0.7%), and (**f**) PPO/GO (0.9%) membranes.

The surface topography of the developed PPO-based membranes was additionally studied by AFM to confirm the surface SEM data (Figure 9).

The surface of all membranes was demonstrated to have a typical nodule structure. The AFM data corroborated the surface SEM micrographs that the introduction of the GO particles into PPO resulted in higher roughness. The GO particles were observed on the surface for modified membranes, and their amount increased with an increase in modifier concentration with respect to the PPO weight. Based on AFM, the surface roughness parameters in terms of average roughness (Ra) and root-mean-squared roughness (Rq) were calculated (Table 1).

The data in Table 1 demonstrate that the PPO/GO (0.1%) and PPO/GO (0.3%) membranes have comparatively the same values of surface roughness within the margin of error, compared to the pristine PPO membrane due to a low GO content in the polymer matrix. Notably, the difference in the surface roughness values (Ra and Rq) of these membranes (PPO, PPO/GO (0.1%), and PPO/GO (0.3%)) did not exceed even 0.8 nm [53]. Only the introduction of GO above 0.5 wt.% significantly increased surface parameters, making it possible to clearly evaluate the contribution of the modification to the change in the membrane surface. The PPO/GO (0.9%) membrane has the highest surface roughness characteristics (Ra = 5.7 nm and Rq= 7.9 nm) compared with other membranes (also confirmed by SEM, Figure 8f), indicating the largest number of GO particle agglomeration on the membrane surface. The increase in surface roughness provides a large effective surface area to contact with the feed components, which is one of the factors resulting in the facilitated sorption and faster penetration of substances. This results in improved modified membrane permeability, which is in agreement with the pervaporation data (Figure 3a) [51].

Mass transfer in pervaporation through a membrane is described by the “solubility-diffusion” mechanism, where the main stages are selective sorption of the feed components on the membrane and their diffusion through the membrane (rate defining stage). In this regard, parameters such as a swelling degree in water and EG and contact angles of water for membranes were investigated to estimate the component transport through the membranes and to assess the changes in the hydrophilic–hydrophobic properties [54] during modification of the PPO-based membrane (Table 2).

PPO is a relatively hydrophobic polymer with a high contact angle of water. The contact angle of water for the pristine PPO membrane was equal to 89°; the corroborating values for the PPO membrane were obtained in the previous studies [23,41,43]. The value of contact angle of water for the PPO/GO (0.1%) membrane was equal to the PPO membrane (89 ± 2°). For the PPO/GO (0.3%) membrane, the contact angle of water slightly decreased to 87°, and the difference with the PPO membrane in the value of the contact angle did not exceed 2°, which was due to the low modifier contents in the membrane. It is also worth noting that these concentrations did not change significantly the morphology and the surface parameters of the PPO membrane (confirmed by SEM and AFM data, Figure 8 and Figure 9). The contact angle of water for the modified membranes with over 0.5 wt.% GO decreased considerably with an increase in the modifier content in the PPO matrix, indicating surface hydrophilization. The introduction of GO-associated functional groups improved the hydrophilic properties of the PPO membrane because of the migration of hydrophilic (oxygen-containing) groups to the top of the membrane surface, causing improved permeation flux and water permeance for the modified membranes (Figure 3a,c) [55,56]. The water contact angle values are in agreement with the surface roughness parameters (Table 1). The same effect of increasing the surface roughness and decreasing the contact angle values was previously observed for hydrophobic polyimide hollow fiber membranes and polyamide/poly(acrylonitrile) (PAN) membranes modified by GO [57,58]. Based on the swelling experiments in water and EG, the sorption and diffusion properties of the PPO-based membranes were determined. The introduction of GO into the PPO matrix enhanced the swelling of the membranes in both components. Additionally, the swelling increased with the rise in the modifier content (Table 2). This effect could be associated with the structure of GO possessing oxygen-containing groups, providing a higher affinity of the modified membranes to polar compounds compared to the pristine PPO matrix [24]. Mass transfer of the components of the separated mixture largely depends on the affinity of the penetrants to the membrane, and an increase in the swelling of the membranes in water and EG increased the permeability of the membranes, especially with the higher GO content in the PPO matrix (Figure 3a) [25]. It is also worth noting that the swelling in EG for the PPO-based membranes was slightly higher than that in water (Table 2). This indicates the enhanced membrane interaction with EG, rather than with water, and the formation of transport channels for the water penetration [41].

**Figure 10:**
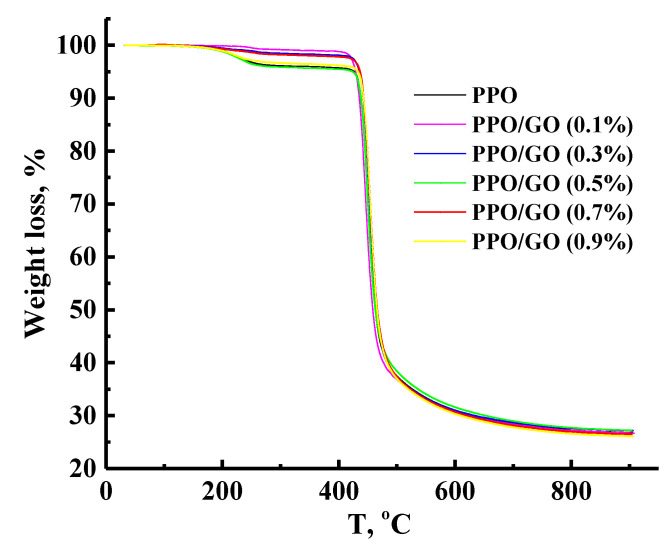
TG curves for the dense PPO and PPO/GO membranes.

The thermal stability of the PPO-based membranes was investigated by TGA. Figure 10 demonstrates thermogravimetric (TG) curves (weight loss) of the samples under heating from 30 to 950 °C.

It was shown that all PPO-based membranes were heat-resistant over a wide temperature range as was demonstrated previously [44]. The TG patterns for the membranes based on pristine PPO and the GO composites are similar and demonstrate two stages of weight loss. In the first stage up to 400 °C, there is a relatively small weight loss of up to 4 wt.% for all membranes. This observation is connected to the residual solvent evaporation and the elimination of low molecular weight impurities [44]. The second range refers to the area from 400 °C. The weight loss of up to 70 wt.% is related to the decomposition of polymer chains. The residual mass of all samples was ~27 wt.%. It should be also noted that GO is highly heat-resistant with combustion temperature in the range of 550–616 °C, depending on the particle size [59]. Based on the TGA data, no changes in the TG curves were observed in the membranes, possibly due to the low level of GO concentration in the membrane matrix. The modification did not significantly affect the thermal stability of the PPO-based membranes.

#### 3.2.2. The Study of the Supported Membranes

**Figure 11:**
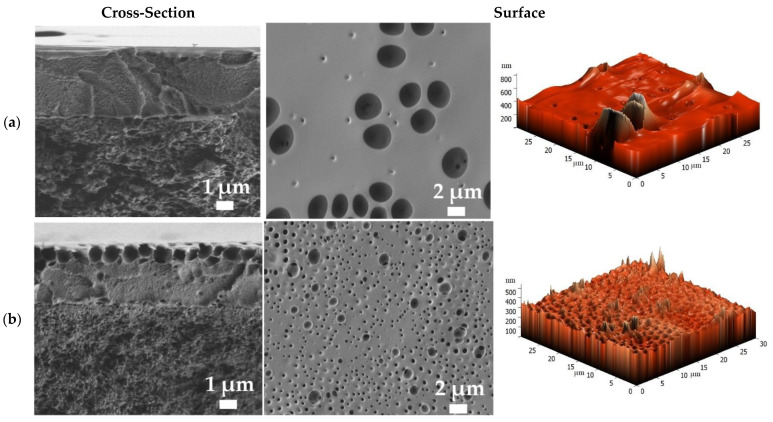
SEM surface and cross-sectional micrographs and AFM images of the supported (**a**) PPO/MFFC and (**b**) PPO/GO (0.7%)/MFFC membranes.

To confirm suitable adhesion and to evaluate the thickness and the surface parameters of the selective dense layer, deposited onto the porous MFFC substrate, the developed supported membranes were investigated by SEM, AFM, and contact angle measurements. It was found that the values of contact angles of water for the supported PPO/MFFC and PPO/GO (0.7%)/MFFC membranes were equal to those for the dense membranes (89 ± 2° and 85 ± 2°, respectively, Table 2), which also confirmed the uniformity and continuity of the thin selective layer on the porous MMFC substrate. The SEM micrographs and AFM images of the PPO/MFFC and PPO/GO (0.7%)/MFFC membranes are presented in Figure 11.

The cross-sectional SEM micrographs of the supported membranes clearly demonstrate two regions: (1) the porous MFFC substrate, and (2) a thin, dense selective layer based on the PPO and PPO/GO (0.7%) composite. The thickness of the thin, dense selective layer based on the PPO or PPO/GO (0.7%) composite was determined to be 3 ± 0.2 µm. Continuous and homogeneous adhesion of the thin, dense layers to the surface of the porous MFFC substrate is also observed. There was no leakage of the polymer solution and composite into the pores of the substrate. Notably, the supported membranes had rounded cavities on the surface of the thin selective layer, as the dense membranes did (Figure 8). These cavities were not hollow (confirmed by SEM cross-sectional micrographs) and they increased in number with a decrease in size, when GO was introduced into the PPO matrix. The same effect of the formation of additional surface cavities was observed during PPO modification with heteroarm star-shaped macromolecules and ionic liquid in the works [25,47]. This effect, together with the hydrophilization of the membrane surface during the modification, may contribute to increased permeability of the modified membrane (Figure 4). Based on AFM images, the average roughness (Ra) and root-mean-squared roughness (Rq) for the supported membranes were calculated (Table 3).

It was found that the deposition of the thin PPO and PPO/GO (0.7%) layers onto a porous MFFC substrate led to a significant increase in the surface roughness compared to the dense membranes (Table 1). However, the surface parameters of the PPO/GO (0.7%)/MFFC membrane are significantly higher compared to the PPO/MFFC membrane, due to the presence of GO nanoparticles in the thin selective layer. The increased surface roughness of the modified membrane can also be attributed to the formation of a larger number of cavities on the surface, causing the rise in permeability for the modified membrane.

### 3.3. Membrane Performance Comparison in Pervaporation Separation of EG/Water Mixture

The performance of polymeric membranes described in the literature for pervaporation dehydration of EG was compared with the developed dense and supported membrane based on the PPO/GO (0.7%) composite in terms of permeation flux and separation factor (Table 4).

The developed dense PPO/GO (0.7%) membrane had higher permeation flux in pervaporation dehydration of EG (10 wt.% water), even under the separation at room temperature, compared to the membranes presented in the works [7,23]. However, the separation factor (4491) was lower compared to the PPO/heteroarm stars with C_60_ (5%) center membrane [23]. The polybenzimidazole/polyetherimide dual-layer hollow fiber membrane had increased permeation flux and decreased separation factor compared to the dense PPO/GO (0.7%) membrane, but its transport properties were measured at a much higher experimental temperature (60 °C) [13]. As for the developed supported PPO/GO (0.7%)/MFFC membrane, it has an enhanced separation factor (4082) compared to all presented supported membranes, including the commercial ones [61]. Furthermore, it should be noted that permeation flux for the PPO/GO (0.7%)/MFFC membrane is lower compared to the membranes described in the works [10,11,60,61], but this is related to the difference in the temperature of the experiment. The permeation flux of the developed membrane can be significantly increased by the use of higher temperatures; the membrane’s high thermal stability was confirmed by TGA (Figure 10).

**Table 4:** Comparison of transport properties for the membranes in pervaporation dehydration of ethylene glycol (10 wt.% water).

Membrane	Temperature, °C	Permeation Flux, g/(m^2^·h)	Separation Factor (*β*)	Reference
Dense membranes				
PPO/GO (0.7%)	22	78	4491	This study
PIM-1 (polymer of intrinsic microporosity)	30	51	39	[7]
PPO/heteroarm stars with C_60_ center (5%)	50	21	11,240	[23]
Polybenzimidazole/polyetherimide dual-layer hollow fiber membrane	60	115	1763	[13]
Supported membranes				
PPO/GO (0.7%)/MFFC	22	180	4082	This study
Polyethylenimine-poly(acrylic acid) (PEI/PAA) complex/PA	22	12	415	[12]
PVA/buckypaper	30	26	802	[4]
1-butyl-3-methylimidazolium tetrafluoroborate-PVA (70/30)/buckypaper	30	102	1014	
Chitosan/PS	35	300	104	[10]
PVA/PS	60	360	987	[60]
Polyelectrolyte complex/GO (3 wt.%)/PS	60	961	1191	[11]
GFT1001 (PVA/PAN)	75	244	1116	[61]
GFT1000 (PVA/PAN)	75	56	141	
GFT1510 (PVA/PAN)	75	1700	591	
DEG167 (PVA/PAN)	75	500	991	

Thus, it was demonstrated that supported PPO/GO (0.7%)/MFFC membrane (modified with 0.7 wt.% GO) had the optimal transport characteristics in pervaporation dehydration of EG compared with literature-described polymeric membranes, and is promising for application in industrial EG regeneration processes.

## 4. Conclusions

In this study, novel dense and supported mixed matrix membranes based on poly(2,6-dimethyl-1,4-phenylene oxide) modified with graphene oxide nanoparticles were developed for improved pervaporation dehydration of ethylene glycol. The introduction of graphene oxide (0.1–0.9 wt.%) into the PPO matrix improved permeation flux of the dense membranes with a slight decrease in selectivity with respect to water, compared to the unfilled PPO membrane in pervaporation dehydration of EG in a wide concentration range (10–90 wt.% water). These observations were related to the changes in the structure and physicochemical properties: the formation of a more amorphous structure of the membrane, rougher inner and surface morphology, and surface hydrophilization (confirmed by various techniques—FTIR, NMR, SEM, AFM, swelling and contact angle measurements). The dense PPO/GO (0.7%) membrane had the optimal transport characteristics: the highest permeation flux of 78–470 g/(m^2^·h), and 99.3–96.2 wt.% water in the permeate in pervaporation dehydration of EG (10–90 wt.% water). To improve the permeability of this dense membrane for promising industrial applications, the supported membrane consisting of a thin selective layer based on the PPO/GO (0.7%) composite deposited onto the porous MFFC substrate was developed. This modified supported PPO/GO (0.7%)/MFFC membrane demonstrated ca. 15 times higher permeation flux, maintaining high selectivity (99.8–99.6 wt.% water in the permeate), compared to the dense PPO membrane in pervaporation dehydration of EG (10–30 wt.% water). Thus, the supported PPO/GO (0.7%)/MFFC membrane is promising for industrial application in EG dehydration processes.

## Figures and Tables

**Table 1:** Surface roughness parameters of the dense PPO and PPO/GO membranes.

Membrane	Surface Parameters	
	Ra, nm	Rq, nm
PPO	3.8 ± 0.2	6.6 ± 0.2
PPO/GO (0.1%)	3.3 ± 0.5	5.9 ± 0.5
PPO/GO (0.3%)	3.5 ± 0.5	5.8 ± 0.5
PPO/GO (0.5%)	5.2 ± 0.5	7.2 ± 0.5
PPO/GO (0.7%)	5.5 ± 0.5	7.5 ± 0.5
PPO/GO (0.9%)	5.7 ± 0.5	7.9 ± 0.5

**Table 2:** Contact angles of water and swelling degrees of the dense PPO and PPO/GO membranes.

Membrane	Contact Angle of Water, °	Swelling Degree, %	
		Water	Ethylene Glycol
PPO	89 ± 2	1.4	3.4
PPO/GO (0.1%)	89 ± 2	1.8	3.5
PPO/GO (0.3%)	87 ± 2	2.7	3.5
PPO/GO (0.5%)	86 ± 2	3.5	4.4
PPO/GO (0.7%)	85 ± 2	6.4	7.1
PPO/GO (0.9%)	83 ± 2	6.6	7.7

**Table 3:** Surface roughness parameters of the supported PPO/MFFC and PPO/GO (0.7%)/MFFC membranes.

Membrane	Surface Parameters	
	Ra, nm	Rq, nm
PPO/MFFC	33.5	68.1
PPO/GO (0.7%)/MFFC	54.6	82.7
